# Rethinking Norm Psychology in Good Company

**DOI:** 10.1177/17456916231187398

**Published:** 2023-08-02

**Authors:** Cecilia Heyes

**Affiliations:** Department of Experimental Psychology & All Souls College, University of Oxford

In the target article, I tried to change some norms—to persuade my social group that we should define and investigate norm psychology in a new way. This challenge to existing norms could have met with punishment—harsh attempts to get the deviant back into line. Instead, the commentators offer a richly constructive set of critical and elaborative thoughts, for which I am sincerely grateful. I cannot do them justice in 5,000 words, but I will respond as best I can under the three headings used in the target article: explanatory target, evidence, and model.

## Explanatory Target

I argued that it would be helpful to shift the focus of norm psychology from rules to behavior. Westra and Evans agreed, but they suspect that rules haunt my characterization of norm psychology as the set of psychological processes responsible for compliance, enforcement, and commentary behavior. “After all, what is compliance, if not compliance with a rule? What does one enforce, if not rules? What is normative commentary about, if not compliance with and violations of rules?” (p. 67). Great questions. I agree with Westra and Evans that “the behaviors that enact compliance, enforcement, and commentary are nearly as varied as the entire range of human behavior itself” (p. 67), but I do not see this variation as an obstacle to a rule-free, behavioral target for norm psychology, a target centered on observable action and agnostic about underlying psychological processes. As I see it, an agents’ compliant behavior is the subset of all their behavior that is enforced (or, as Westra & Evans nicely described it, “maintained”) by the positive and/or negative reactions of other members of their social group. In a complementary way, agents’ enforcement behavior is the subset of all their behavior that through the delivery of rewards and punishments, functions to maintain compliant behavior in other group members. And normative commentary is about what ought and ought not to be done. In academic circles and the wider world, commentary behavior is typically linguistic and often involves the articulation of rules. However, in common with Westra and Evans, I do not regard even these features as a guarantee that commentary behavior is generated by mental rules.

Norms are conventionally understood to be properties of social groups. Evans and Westra honored this convention by defining “normative regularities” as properties of social groups. I honor the convention, too. I suggested that it is a strength of my behavior-based characterization of norm psychology (note that I am not offering a definition of “norms”) that it “leaves room for us to think of norms as rules—spoken or written statements—or ‘standards’ that have been inferred from compliance, enforcement, and commentary behavior by people within a society or by observers from outside” (p. 22). But I still think people who want to understand the processes inside individual heads that support group-level norms—norm psychologists—should set their sights on behavior, the traditional explanatory target of psychology, rather than rules. This orientation does not imply that normative behavior can be identified without reference to a social group or that norms, understood as group-level rules, are reducible to individual behavior.

Germar and Mojzisch were also firm in regarding norms as “a group-level phenomenon.” They did not see conflict between the group dependence of norms and my behavioral characterization of norm psychology, but they drew attention to an important gap that needs to be filled. If the explanatory target of norm psychology is the behavior of individuals (compliance, enforcement, and commentary), how can it connect with group-level properties of norms? For example, how can it inform and be informed by research on the life cycle of norms—their emergence, stabilization, change, and removal?

A rules-based characterization of norm psychology is at risk of hiding this kind of explanatory gap by using the same term, “rule,” to characterize the group-level explanandum and the individual-level explanans. The duplicate terminology could seduce one into thinking that a group-level rule against spitting is explained by rules against spitting on the heads of group members. Nonnormative cases make clear that this reduction will not work. The size of my group has an impact on what I and other members of the group believe is the size of the group and vice versa—if we thought the group was smaller, we might leave—but our beliefs about group size are radically insufficient to explain the size of the group. For anything resembling a satisfactory explanation of group size—and group norm content—it is necessary to consider not only what is inside individual heads but also the group’s ecology, history, and institutions.

A behavior-based characterization of norm psychology reminds researchers that hard work is needed to connect features of groups to features of individuals. This challenge confronts all scientists seeking to explain “emergence”—systems with features that are not found in their components. I doubt that this core challenge is greater for cultural-evolutionary than for nativist norm psychology or with a behavior-based rather than a rules-based explanatory target, but as Germar and Mojzisch pointed out, the challenge still needs to be met. I cannot do that here and now—such integration is more naturally seen as a fruit than a seed of cultural-evolutionary norm psychology—but I have two thoughts about the life-cycle case highlighted by Germar and Mojzisch. First, I am not sure why they believe that a behavior-oriented norm psychology is in a better position to explain the stabilization/persistence phase of the life cycle of norms than the change phase. It seems that in both cases, understanding the psychological processes that mediate compliance, enforcement, and commentary would make a significant contribution, but it would also be necessary to appeal to suprapsychological factors—ecological, economic, institutional, political—to get a satisfactory explanation of why at particular times and in particular societies some kinds of norms survive while others perish. Second, I suspect that the psychology of individual differences will prove especially useful in explaining both stabilization and norm change. When discussing the poverty–wealth scheme, I noted that variation between groups and individuals within a society is an important source of evidence neglected by nativist norm psychology. This point is underlined by Germar and Mojzisch’s own work, including their study that showed people with higher basal testosterone are more receptive to minority positions (Germar & Mojzisch, 2021). Perhaps, then, to make norm psychology more useful for the broader project of explaining norms, researchers need to emphasize that the extent of compliance, enforcement, and commentary varies among individuals within societies, and it is important to trace the psychological sources of this variation because it has major group-level consequences.

Birch and Theriault thought that my cultural-evolutionary framework said far too little about feeling, about “normative motivation,” “normative phenomenology,” and “affective pressure” to conform to norms. I agree. I neglected these topics because they were not central to Sripada and Stich’s (2006) project, to which I was responding, and they lie outside my areas of expertise. I am a psychologist of thinking rather than feeling. But I do not doubt the importance of the questions highlighted by Birch and Theriault: Is it the case that across cultures, the phenomenology of obligation is distinct from the phenomenology of desire/aversion? Whether universal or culture-specific, what are the psychological processes and evolutionary pressures that yield a distinctive feeling of obligation? What role, if any, does this feeling play in generating normative behavior—compliance, enforcement, and commentary? There is a natural affinity between the constructivist theory of emotion, cited and advanced by Theriault, and cognitive-gadgets theory (Heyes, 2018a). This affinity was recently developed into a set of testable hypotheses about “the cultural evolution of emotion” (Lindquist et al., 2022). Therefore, I hope that Theriault, Lindquist, and other experts on emotion will find a use for my ideas about a normativity gadget in developing a more complete account of the cultural evolution of normativity.

Although fascinating, I do not believe that the phenomenology of obligation should replace or rank alongside normative behavior in the conceptualization of the explanatory target of norm psychology. If norm psychology is to be a successful empirical science, its explanatory target needs to be clear for all to see. On some accounts of introspection, phenomenal experiences are observable, but complex feelings, such as feelings of obligation, are notoriously difficult to investigate even in adult humans, let alone in infants, children, and nonhuman animals. Addressing fellow members of our social group (centered on readers of *Perspectives on Psychological Science*), Birch cleverly evoked the feeling of “affective pressure toward following a norm” with his “Contract” example, but there is ambiguity even within our small world. My intuition is that in “Contract”-like cases, I experience not a norm-specific feeling of obligation but a generalized feeling of conflict—an aversive state like the one I get when, under time pressure, I cannot decide whether to switch queues in the supermarket. (Sorry, I’m British—queues matter.) Sensitive empirical methods would be needed to find out whether my intuition or Birch’s is more typical of a culture or indeed of humanity in general. That hard empirical work is well worth doing, but until more of it is done, I think it would be unwise to define norm psychology in relation to the phenomenology of obligation or to constrain theorizing based on assumptions about its character.

Moore and Monso asked me to clarify whether on my view of norm psychology, there could be normative animals. My answer is yes. Using Monso and Moore’s framing in terms of necessary and sufficient conditions, I take language to be necessary for the development of commentary and commentary to be necessary for explicit normativity, but “compliance and enforcement are necessary and cosufficient” (p. 52) for implicit normativity. Thus, researchers would have evidence of normative behavior in nonhuman animals if, for example, they found that new members of a group of vervet monkeys adopted the food preferences of existing members (compliance; Van de Waal et al., 2013) *because* existing members rewarded group-typical feeding behavior and/or punished alternative feeding behavior (enforcement). Of course, on my lean account of implicit normativity, one would not need evidence that the enforcers represent their behavior as group-typical or intend their positive and/or negative reactions to make new group members behave in a group-typical way.

## Evidence

My poverty–wealth scheme suggests that hypotheses about the contributions of nature, nurture, and culture to a psychological capacity—including the capacity for normative behavior—should be tested by asking whether the developmental environment provides too little (poverty) or at least enough (wealth) usable information to explain variation across (a) time points in development, (b) groups and individuals within a society, (c) human societies, and (d) species. I was delighted that three of the commentaries commended the poverty-wealth scheme (Germar & Mojzisch; Richerson & Gavrilets; Taumoepeau) and no one offered a challenge. This bodes well given that the primary purpose of the target article is to stimulate the development of a norm psychology based not on the accumulation of confirmatory evidence but on contrastive empirical testing.

Drawing on Marr (1982), Vogel and Lockwood underlined a crucially important point in an elegant way: Normative behavior needs to be explained with reference to its evolutionary function (computational level), psychology (algorithmic level), and neurobiology (implementation), and therefore cognitive neuroscience is an important source of evidence for norm psychology. They also suggested that like (other) mechanisms of social learning, the processes mediating normative behavior may be domain-general at the computational level but domain-specific at the implementation level. I am convinced they are right about this by their data showing that “the same algorithms (associative learning) can guide both social and nonsocial learning but that the implementation is realized in partially distinct brain areas” (p. 54). Therefore, I should clarify: When I argue in the target article that implicit normativity is based on domain-general processes, I am referring to the algorithmic level, not the implementation level. The domain-general algorithms that mediate learning to comply and to enforce are likely to be centered in brain regions that have easy access to information from other agents, and these regions may vary across species.

On one reading, the main thrust of Richerson and Gavrilets’s commentary is like that of Vogel and Lockwood. Correcting the focus of the target article on behavioral data, they argued that neurobiological data are a valuable resource for norm psychology. I can only endorse this message and thank Richerson and Gavrilets for some fine examples from behavioral neuroscience. On an alternative reading, Richerson and Gavrilets have a bracingly radical agenda. They envisaged an “evolutionary neuroscience” that combines “top-down evolutionary functionalism” to characterize adaptive problems with “bottom-up neurobiology” explaining how primate brains are organized to solve these problems—and, apparently, cuts out the psychological middleman. In Marr’s terms, such an evolutionary neuroscience would go straight from the computational level to the implementation level, ignoring the algorithms. In the case of norms, it would replace norm psychology with norm neuroscience. Marr (1982; Peebles & Cooper, 2015) made a strong case that such a two-level strategy is unlikely to be successful in explaining behavior. Rather than rehearse his arguments, I will quietly point out that—with references to anger, fear, pleasure, aversion, hunger, and thirst—there is plenty of psychology in Richerson and Gavrilets’s commentary. Like many cultural evolutionists, they use folk psychology while leaving scientific psychology unmined, and I think that is a missed opportunity. Richerson and Gavrilets are right—norm psychology needs neurobiology. But also, cultural evolution needs cognitive science (Heyes, 2018b).

Taumoepeau’s reflections on the kinds of evidence needed by norm psychology are rich, detailed, and immensely helpful. (For example, she indicated how psychologists could address Moore & Monso’s call for more information about the impact of language on the development of normative behavior.) At the most general level, Taumopeau’s discussion of cross-cultural variation in developmental sequences makes clear, in a way I did not, that the four sources of variability can be combined to provide additional resources for hypothesis testing. For example, compared with a nativist hypothesis about the early development of compliance, a cultural-evolutionary hypothesis might predict a particular pattern of variability in the extent to which the developmental sequence (Source 1) varies across individuals or groups within a society (Source 2), between human societies (Source 3), and/or across species (Source 4). In a complementary way, Taumoepeau made a compelling case for using cross-cultural data to lead inquiry about the psychological mechanisms underlying normative behavior. Her authoritative vision is one in which researchers in the West relinquish the habit of basing hypotheses on their own introspection and moral traditions and only later, if at all, asking whether they fit other cultures. Instead, the inquiry would be rooted in cross-cultural research on norm-relevant practices, such as fluid collaboration, caregiver interactions, and conversational styles. This work would not only provide a test bed but also inspire the hypothesis to be tested. Taumoepeau and I are not in perfect accord. I am not the kind of social constructivist who rejects computationalism, and perhaps as a corollary, I find the concept of “internalization” too vague to be helpful, but I am impressed by her vision and hope it will be shared increasingly among norm psychologists.

Four commentaries that focused on my cultural-evolutionary model (see below) rather than methodological issues nonetheless made valuable points about evidence. Kish Bar-On and Lamm argued that normative behavior evolved in social groups that were often hierarchical and unstable. Therefore, although it is convenient to use simple stable groups in laboratory experiments on explicit normativity, it would be wise to check that results generalize to more complex group structures.

In a more radical intervention, Pain argued not only that archaeological data can inform about the evolution of norm psychology but also estimates using stone tool data that a norm gadget first emerged sometime in the Acheulean period (1.7 million years ago to 300,000 years ago) in *Homo heidelbergensis* and perhaps later variants of *Homo erectus*. As Pain discussed with great subtlety elsewhere (e.g., Pain, 2021), estimates of this kind depend on big assumptions about the kinds of psychological process required to produce stone tools, and some of these assumptions depend, in turn, on theoretical frameworks that are more popular in cognitive archaeology than in cognitive science as a whole (e.g., 4E). These reflections are not cautionary. In my view, along with Pain’s commentary, they should encourage a wider range of cognitive scientists to dive into cognitive archaeology. However, I am not yet sure how much work archaeological data can do within the poverty–wealth scheme. Given that the scheme is designed to promote contrastive rather than confirmatory testing, a key question for me is whether emergence of normative competence during the Acheulean makes it more or less likely to be based on a cognitive gadget rather than a cognitive instinct.

In their title and footnote, Schmidt, Vaish, and Rakoczy likened me to “Inspector Gadget,” who “solves complicated cases, performs heroic acts, furthers the general good—but sometimes does so despite (or because of?) neglecting some options” (p. 70). I think that is fair and, like the tone of the whole commentary, very kind. I will take “Inspector Gadget” for my headstone. I want to thank Schmidt et al. for their kindness and to state more plainly than I did in the target article my admiration for their work and, more generally, for the corpus of research that seeks to explain normativity within a constructivist view of the mind and with reference to “shared intentionality.” This work is rigorous, creative, and always illuminating.

The problem, as I see it, is that the shared-intentionality “third way”—the theory that normative behavior is rooted in a rich, genetically inherited, human-specific but not norm-specific psychological capacity—is being developed in isolation. Specifically, it is not being tested against alternatives. Instead, the empirical work relating to shared intentionality uses a confirmation strategy. For example: (a) Shared-intentionality theory predicts behavior X in condition Y but not condition Z. (b) X is observed in Y but not Z. (c) Therefore, shared intentionality theory is confirmed. Yes, many of the developmental studies control “for regularity and familiarity of the actions” (p. 70) observed by infants but rarely as part of an explicitly contrastive testing strategy. For example: (a) Shared-intentionality theory predicts X in Y but not Z, whereas a specified alternative theory (postulating richer or leaner genetically inherited resources) predicts X in Z but not Y. (b) X is observed in Y but not Z. (c) Therefore, shared-intentionality theory is supported relative to the specified alternative theory. As illustrated in the target article’s discussion of “normative protest,” data collected via a confirmation strategy can often be just as readily explained by an alternative theory and therefore do not take the field any closer to explanation (Press et al., 2022). Shared intentionality is a highly elaborated theory with a large amount of associated data. It is an inviting prospect because it offers an encompassing view with lots of illustrative examples. But if the data, individually and as a whole, are equally compatible with an alternative theory, researchers should resist the invitation; reserve judgment until the results of contrastive testing are in.

Contrastive testing is easier when the alternative theories are alike in their fundamental assumptions (e.g., both computationalist) and far apart in their substantive claims (e.g., about domain-specificity). Consequently, as they are currently formulated, it would be easier to test the nativist and cultural-evolutionary accounts of norm psychology against one another than to test either against shared intentionality. Nonetheless, I would be delighted to see shared intentionality give up its glorious isolation and enter the ring.

I wrote at the beginning of this section on evidence that none of the commentaries challenged the poverty-wealth scheme. However, a comment of Sterelny’s has the potential to become a call for extension to the scheme. If normative cognition is generally adaptive and if cultural selection is generally less likely than genetic selection to bring about adaptive processes of normative cognition, then—along with poverty of the stimulus—the adaptiveness of a norm-specific process is prima facie evidence of a role for genetic selection in its evolution. Sterelny advanced this kind of argument, but in relation to norm content rather than norm-specific processes and in a way that militated against both cultural and genetic selection of individual norms. I doubt that the argument could be extended to norm-specific processes in a way that would favor genetic over cultural selection. However, if it could, the poverty-wealth scheme would need an additional component relating to adaptiveness.

## Model

In the final part of the target article, I outlined a cultural-evolutionary model suggesting that human normative behavior depends on implicit psychological processes that are domain-general, predominantly genetically inherited, and shared with many other animals plus a normativity gadget—a set of explicit psychological processes that are domain-specific, shaped by cultural selection and distinctively human.

Sterelny doubted that the explicit processes are domain-specific in the right way to make them a cognitive gadget. In a characteristically incisive challenge, he said that “Normative cognition seems to be cognition about a distinctive domain, not a distinctive way of thinking about that domain” (p. 43). And, of course, Sterelny may be right. At the algorithmic level (Vogel & Lockwood), people might reason about norms in the same way as they reason about, for example, causality. As far as I am aware, the only directly relevant, rigorous psychological research pits a domain-specific “pragmatic reasoning schemas” account of deontic reasoning (Cheng & Holyoak, 1985) against domain-general alternatives, most prominently the “mental models” theory (Johnson-Laird, 1983). The former view suggests that people reason using learned “knowledge structures” consisting of generalized sets of rules defined in relation to specific domains, such as permission, obligation, and causality. These rules, such as “If the action is to be taken, then the precondition must be satisfied” or “If the precondition is satisfied, then the action may be taken,” are domain-specific but abstract, and the rules within a schema interact in a distinctive way to produce behavioral outcomes.

I am persuaded by the evidence for pragmatic schemas, but I am not a specialist in this highly technical field; few specialists would claim outright victory for either side, and the battle has mostly been fought over the performance of Western people in the Wason selection task. A cultural-evolutionary perspective clearly implies that future research on explicit normativity should encompass many cultures and reasoning tasks. My hunch that this future research should look for processes that are domain-specific in their mode of operation depends not only on pragmatic schemas theory but also on resemblances between literacy—a paradigmatic cognitive gadget—and explicit normative competence. They both involve complex computations with interpretive and regulative functions; a literate person can both read and write, and a person with explicit normative competence can both detect and generate normative behavior. The target article surveyed evidence that, like literacy, the development of explicit normativity depends on effortful cultural learning and yet becomes automatic with intensive practice. Like reading, which interferes with performance on the color-naming Stroop task, normative expertise cannot readily be switched off (cf. Birch). There are marked individual differences in competence (e.g., dyslexics and psychopaths), but academic experts on literacy are no more likely to be speed readers than moral philosophers are to be good people.

At first, I thought that Knobe was on the same page as Sterelny. He seemed to be raising the possibility—with a fine evidence base and precious lack of dogmatism—that none of the thinking behind normative behavior is domain-specific in a way that could make it a gadget. However, after reading Knobe’s commentary more closely and going back to his previously published work, I now believe that he and I are kindred spirits. This was concealed in the target article by my use of terms such as “descriptive-prescriptive conflation” to characterize evidence that children and adults are influenced in their judgments of what is right by information about what is statistically normal and vice versa. “Conflation” may have implied commitment to the idea that people have two distinct kinds of representation that sometimes get tangled up together—statistical representations fitting the case of an all-black tiger and prescriptive representations fitting the case of the steak-eating coworker. In fact, I am very open to Knobe’s suggestion that people have domain-general, “hybrid statistical/prescriptive representations that represent both how things are and how they ought to be” (p. 61). Recall that my cultural-evolutionary account “is not committed to particular modeling strategies [but] is committed to the view that mature human normativity is (a) rooted in implicit psychological processes that, however they are modeled, do many jobs in many species and (b) distinguished from nonhuman normativity by cognitive and motivational features that are culturally learned” (p. 24). Accordingly, if there are hybrid statistical/prescriptive representations, I would see them as the bedrock of domain-general, implicit normativity and expect to find them in nonhuman animals. The stuff of explicit normativity is the additional processes that allow humans sometimes to distinguish descriptive from prescriptive norms—to pull apart the hybrid representations or to go beyond them. Like Knobe, who wrote compellingly elsewhere about the importance of social learning (e.g., Bear & Knobe, 2017), I see this domain-specific capacity as crucially dependent on cultural learning.

There, I have done it again! I distinguished implicit from explicit processes, when Germar and Mojzisch advised me to drop “the dead weight of the dual-system assumptions” on my cultural-evolutionary model. In a sympathetic way, they pointed out several genuine risks of “dual system” or “dual process” thinking. I refer the reader to a previous forum in *Perspectives on Psychological Science* for detailed discussion of the risks and insight into why many psychologists—including me—are willing to court those dangers because they believe that the dual-process framework captures something real and important about human minds (Evans & Stanovich, 2013a, 2013b). Two points are worth mentioning here. First, I would be appalled if the dual-process framework made hypotheses difficult to test—or as Germar and Mojzisch put it, to “falsify”—but I doubt that is the case. Like most “metatheories” (Evans & Stanovich, 2013b), the dual-process framework cannot be tested directly and holistically, but the hypotheses that it frames and inspires can be subjected to contrastive testing if they are fashioned with that aim in mind. I tried to illustrate this in the target article with an extended discussion of children’s normative protest. Second, I see the necessity to explain “the transition between the two qualitatively distinct systems” as a feature rather than a bug in my dual-process model of normativity. Nativist accounts of psychological functions tend to dodge the need to explain cognitive development by suggesting that the critical competences are all there “in the genes” from the start. In contrast, a cultural-evolutionary perspective makes clear that explaining the transition from implicit-only to implicit-plus-explicit processes and the subsequent interaction between implicit and explicit processes is a focal challenge for norm psychology.

The target article suggested that language is crucial in the transition from implicit-only to implicit-plus-explicit normativity but did not offer any detailed proposals. Moore and Monso and Pain are right to pick up on this lack of detail, but I would prioritize one of their calls over the other. A mature, cultural-evolutionary account of norm psychology needs to say much more about the role of language in the development of explicit normativity. Scientists need a model of how exposure to normative language promotes the development of distinctively normative thought (and Taumoepeau has some excellent suggestions for sources). But I am not sure a cultural-evolutionary account of normativity needs a matching or complementary theory of the evolution of language. Of course, it would be nice to have a more complete picture, but as far as I can see, explicit normativity could be a cognitive gadget even if language is a cognitive instinct.

Calling for expansion of the cultural-evolutionary model to encompass thinking about groups and group membership, Kish Bar-On and Lamm cannily pointed out that the concept of a human group recurs as often in definitions of norms and norm psychology as the concept of a rule. I would very much welcome such expansion, and I was intrigued by Kish Bar-On and Lamm’s (necessarily) brief survey of evidence that “group thinking,” like norm psychology, is a cognitive gadget. However, as in the case of language (Moore & Monso; Pain), I am not sure that a cultural-evolutionary norm psychology would be threatened by evidence of significant genetically inherited predispositions to think about groups in particular ways. It would reduce a potential source of cultural variation in explicit normative cognition, but given manifest cultural variation in ecology, group structure, and norm content, there are plenty of other sources.

In his commentary and elsewhere (Birch, 2021), Birch argued that human normative thought is a practical skill that evolved as a solution to the challenge of transmitting complex crafts, especially tool-making skills, through social learning. Like Pain, I am drawn to this idea. Birch sees domain-specific normative skill as implicit and genetically inherited, whereas I regard it as explicit and culturally inherited, but I find his core insight compelling: Doing right by your neighbor resembles getting it right with your hands. Therefore, I think that any future developer of the cultural-evolutionary model would be wise to consider whether explicit normative competence is concocted by cultural learning not only from reasoning and mentalizing but also from the psychological processes mediating practical skill.

## Conclusion

Brady and Crockett’s wonderful commentary is in a class of its own. They took the last two sentences of the target article and ran like Olympians. Their tour de force is an analysis of how characteristics of social media—hidden incentives, context collapse, and speed of transmission—are changing normative cognition. They are careful throughout to distinguish effects of social media on normative cognition from effects on norm content and to distinguish fact from speculation. Many of their findings are chilling, but they finish on an upbeat, policy-relevant note: “We hope that increasing attention to how normativity is shaped by culture—in addition to nature and nurture—can pave the way for designing technologies that accelerate the evolution of a norm psychology better adapted for global cooperation.” I share that hope.

## Transparency

*Action Editor:* Daniel Kelly

*Editor:* Interim Editorial Panel
